# Face masks inhibit facial cues for approachability and trustworthiness: an eyetracking study

**DOI:** 10.1007/s12144-022-03705-8

**Published:** 2022-10-06

**Authors:** Listryarinie Ongko Bylianto, Kai Qin Chan

**Affiliations:** James Cook University, Singapore, Singapore

**Keywords:** Social judgments, Trust, Approachability, Face masks, Eyetracking

## Abstract

Wearing face masks during the Covid-19 pandemic has undeniable benefits from our health perspective. However, the interpersonal costs on social interactions may have been underappreciated. Because masks obscure critical facial regions signaling approach/avoidance intent and social trust, this implies that facial inference of approachability and trustworthiness may be severely discounted. Here, in our eyetracking experiment, we show that people judged masked faces as less approachable and trustworthy. Further analyses showed that the attention directed towards the eye region relative to the mouth region mediated the effect on approachability, but not on trustworthiness. This is because for masked faces, with the mouth region obscured, visual attention is then automatically diverted away from the mouth and towards the eye region, which is an undiagnostic cue for judging a target’s approachability. Together, these findings support that mask-wearing inhibits the critical facial cues needed for social judgements.

**Supplementary Information:**

The online version contains supplementary material available at 10.1007/s12144-022-03705-8.

Faces contain critical cues for social judgments. Facial expressions and gaze cues are vital in judging how approachable or trustworthy one is and likely determine future social interactions. Given the Covid-19 pandemic where wearing face masks is mandatory in many settings, how do face masks affect the way we judge other people? Are there hidden social costs that have gone unnoticed? In this research we seek to understand fundamental attentional dynamics on how face masks influence social judgment facets of approachability and trustworthiness.

## The importance of approachability and trustworthiness in social interactions

Approachability and trustworthiness are fundamental aspects of social judgments that facilitate the formation of social bonds. Judging approachability (i.e., approach or avoidance intent) is adaptive because it signals whether a potential interaction partner is a friend or a foe (Calvo et al., 2018; Mattavelli et al., 2012) and marks the beginning of a social engagement (Adams & Kleck, 2005; Elliot, 2008; Willis et al., 2011a, b). Trustworthiness judgement is critical to avoid the dire consequences from over-trusting and the opportunity cost of mistrusting which have been found to implicate key social outcomes from job hiring selection to governmental election and long-term relationship choice (Bzdok et al., 2010; Carrito et al., 2020; Todorov et al., 2013). From the primitive threat perception ensuring safety to the more sophisticated behavioral intent inference, approachability and trustworthiness judgements play pivotal roles in modulating our social behavioral responses in order to interact appropriately with others and achieve an optimal social outcome (Todorov, 2008; Willis et al., 2011a, b).

## Facial expressions as cues for approachability and trustworthiness judgements

People obtain cues to judge approachability and trustworthiness from a variety of sources, such as facial expressions, eye gaze, and body expressions (Sutherland et al., 2015; Willis et al., 2011a, b). Faces are undoubtedly one of the most important sources with the abundant social information contained by the facial features expressing emotions and signaling behavioral intentions (Bzdok et al., 2010; Calvo et al., 2018; Sutherland et al., 2015; Todorov et al., 2013). Furthermore, face evaluation for social judgements has been found to be very efficient, requiring as little as 100 ms (Bzdok et al., 2010; Willis & Todorov, 2006).

From past experiments involving computer modeling of facial expressions and trait judgements, Todorov (2008) found that trustworthiness best-estimated face valence rating and suggested a close relationship with approachability (Oosterhof & Todorov, 2008; Todorov, 2008; Willis et al., 2011a). First impression studies also showed that instinctive responses were geared towards preference for happy faces and people relied on broad and simple facial cues, such as smile, to judge approachability (Batty, 2020; Sutherland et al., 2015). Researchers found that both social judgments use the same facial features (e.g., inverted-V-shaped brows and U-shaped mouth indicating happy faces) and involve the amygdala (Dzhelyova et al., 2012; Mattavelli et al., 2012; Sutherland et al., 2015). Evolutionarily speaking, it is adaptive to associate facial displays of happiness with greater trustworthiness and approachability because they signal friendship and alliance (Calvo et al., 2018).

Notwithstanding the above, a recent study found that while smiling expression, particularly the accompanying mouth shape and bottom lip curving, are related to trustworthy faces, there is a difference in terms of fixation density on the mouth area of happy faces when people make judgments of a face’s happiness and trustworthiness, with a higher score for the former (Calvo et al., 2018; Hermens et al., 2018; Oosterhof & Todorov, 2008). This suggests that there is still a distinction between happiness and trustworthiness inferences, whereas the association between happiness and approachability seems more direct (Batty, 2020; Sutherland et al., 2015; Willis et al., 2011a). Further eye-tracking studies involving face stimuli and various facial expressions also showed that while happy faces had longer dwell time around the mouth region as compared to the eyes, people tend to scan the eyes, nose and mouth region when assessing trustworthy face, and there was no distinct dominant facial region that could signal and predict trustworthiness, which instead relied on an integration of the cues from the various facial features (Eisenbarth & Alpers, 2011; Hermens et al., 2018). Calvo et al. (2018) attributed such face-scanning gaze behaviour to the need to seek expressive congruency from the various facial cue sources when judging trustworthiness, e.g., a smiling expression but unhappy eyes do not signal truthfulness or reliability and thus would not be regarded as trustworthy.

Hence, while a smile, displayed primarily by the mouth region, is diagnostic of happiness, which is a close inference of approachability, a smile may not be distinctively diagnostic of trustworthiness which is likely dependent on the processing of other facial regions, particularly, the eye region which is also a critical cue for face evaluation (Calvo et al., 2018; Eisenbarth & Alpers, 2011; Hermens et al., 2018; Oosterhof & Todorov, 2008).

## Eye gaze as possible cue for approachability and trustworthiness judgements

Besides facial expressions, eye gaze (known more colloquially as “eye contact”) could therefore be an important cue in social interactions. Direct-gaze faces have been found to attract and retain attention (Dalmaso et al., 2017a, b; Mares et al., 2016) and reduce saccadic eye movements (Dalmaso et al., 2017a, b; Ueda et al., 2014). Direct gaze, accompanied by an eyebrow raising, signals trust and an intent to communicate (or an approach behavior), which has a positive effect on trustworthiness (Frith, 2009; Kaisler & Leder, 2017; Todorov et al., 2013). Further, there is strong preference for direct gaze with full frontal view of the eyes when judging trustworthiness, i.e., clear available eye gaze cue is related to higher trustworthiness rating, particularly when processing unfamiliar faces (Kaisler & Leder, 2017). This could be explained from an evolutionary perspective where primates had to adapt and evolve by making sense of others’ intentions through eye gaze cues. In fact, even very young infants can differentiate direct or averted gaze direction and paid attention to the eyes more than the other facial features (Adam & Kleck, 2005; Cavallo et al., 2015; Cui et al., 2019). Cui et al. (2019) further established the linkage of the positive effect of direct gaze to perceived closeness by using implicit association test investigating the potential spontaneous interpersonal closeness signals sent by direct gaze. Such perceived closeness is related to the warmth dimension of social judgements comprising approachability and trustworthiness (Cui et al., 2019; Dzhelyova et al., 2012; Mattavelli et al., 2012; Oosterhof & Todorov, 2008).

In summary, people infer an interaction partner’s approachability and trustworthiness from faces. Both social judgments are correlated, perhaps because they stem from the central involvement of the amygdala and they utilize very similar facial features. However, there are instances where the correlation between trustworthiness and approachability is imperfect, and conceptually, approachability and trustworthiness are not synonymous as well. Gaze cue could also play a role in forming social judgments. In fact, a recent study found that gaze cueing of attention is not affected by mask-wearing (Dalmaso et al., 2021).

## The impact of wearing face masks

Two studies using neutral faces, found that masked faces were rated as *more* trustworthy and more approachable (Cartaud et al., 2020; Lau, 2021). Two other studies using various emotional faces (Grundmann et al., 2021; Marini et al., 2021) however found nonsignificant differences on trustworthiness between masked and unmasked faces. The results from these latter two studies were less interpretable because their analyses were not split by the emotional expression of their facial stimuli. More importantly, none of these four research investigated mediating mechanisms. Our goal here was not to replicate or explain Lau (2021) and Cartaud et al.’s (2020) findings using neutral faces. We surmised that even if we could replicate their findings, we would not be able to explain them because we could not conceive any plausible explanatory mechanisms for their findings. Instead, we chose to use happy faces as stimuli because there is a clear signaling value of happy faces, as mentioned previously, and that makes directional hypothesis conceivable.

We debated over two possible *a priori* directional hypotheses. Perhaps most intuitively, one could posit that mask-wearing inhibits facial expression of happiness by obscuring the smiling mouth region and thus impairing trustworthiness and approachability. We call this the *inhibitory hypothesis*. However, the opposite prediction could also hold true: It is also possible that because facial expression cues of happiness are obscured, perceivers automatically focus more on the eye region and the increased “eye contact” and the gaze cue that is enhanced compensates or even enhances approachability and trustworthiness judgements for masked faces. We call this the *compensatory hypothesis*. The compensatory hypothesis is conceivable because past research has shown that gazes enhanced perceived interpersonal closeness (Cui et al., 2019; Dzhelyova et al., 2012; Mattavelli et al., 2012; Oosterhof & Todorov, 2008), and enhances the intention to communicate (Frith, 2009; Kaisler & Leder, 2017; Todorov et al., 2013).

## The present research

In this research, we attempted to tease apart these two possible hypotheses about the impact of wearing face masks on social judgments in Singapore: Targets who are wearing face masks would either be rated as *less* approachable and trustworthy (inhibitory hypothesis) or they will be rated as *more* approachable and trustworthy (compensatory hypothesis). Both hypotheses make the same prediction that perceivers would look more at the target’s eyes if the target is masked up, but the key is whether the increased focus on the eyes matter. As such, besides having trial-by-trial self-report data on the focus on eye gaze cues (i.e., perceived gaze intensity), we also added eyetracking evidence (fixation and count) to examine visual dynamics as perceivers make judgments of targets’ approachability trustworthiness.

More crucially, the fixation and frequency of looking at perceiver’s eyes may mediate the relationship between mask-wearing and social judgments, and these may help us better tease apart our two competing hypotheses. If greater attention is focused on the eyes predicts a decrease in approachability and/or trustworthiness judgments, then it reveals that participants are looking at something in the eye region – but it may not necessarily be affiliative cues. On the other hand, if greater attention is focused on the eyes predicts an increase in approachability and/or trustworthiness judgments, then it reveals that participants are looking *at* something in the eye region (e.g., affiliative cue) – and they have likely found it. In short, both the direct (path *c*) and indirect effects – particularly path *b* – matter.

## Method

### Participants

Thirty-seven participants (*M*_age_ = 27.22; *SD*_age_ = 6.72; 25 females and 12 males) All participants had normal or corrected-to-normal vision, with 13 wearing contact lens and two using glasses. All participants were of Asian origin. The sample size of 37 participants met the requirement to achieve medium effect size for within-subject experimental design, calculated using G*Power analysis (Faul et al., 2007) based on *F* tests ANOVA Repeated measures, with medium effect size *f* = 0.25.

Participants comprised students from a university in Singapore and the general population, recruited via snowball sampling. Course credits were given to students who needed them; otherwise, no remuneration was given.

### Materials

#### Design

The independent variable was facial masks of target stimuli (masked vs. unmasked). Three self-reported dependent variables (i.e., trustworthiness, approachability, and perceived gaze intensity) and two eye-tracking parameters (i.e., fixation duration and fixation count) were recorded.

#### Apparatus used

A head-mounted Pupil Core monocular 200-Hz eye tracker (PupilLab©), connected to a 13-in MacBook Air recorded eye-tracking data when participants were presented with face stimuli. In addition, an all-in-one Dell 23.8-inch (1920 by 1080 pixels resolution, at a 50–60 Hz refresh rate) computer was used to present the face stimuli and record all non-eye-tracking data.

#### Stimuli

 Facial stimuli comprising 30 young (*M* = 23.82 years, *SD* = 4.18; 15 male and 15 female faces) Chinese ethnicity faces were selected from the validated Tsinghua facial expression database (Tsinghua University, 2020; Yang et al., 2020). Using faces of Chinese ethnicity was appropriate because of Singapore’s predominantly Chinese ethnicity demographic. All faces displayed a happy facial expression (*M* = 4.13, *SD* = 0.29, on a scale of 1 = *least intense* to 5 = *most intense*) and displayed frontal gaze (Yang et al., 2020).

All stimuli were resized to 756 by 945 pixels, corresponding to around visual angles of 17° by 22° and had a white background with consistent illumination. A black face mask was superimposed onto each original unmasked face stimulus to create another set of “masked up” faces. Sample images are found in Online Supplementary Materials (OSM): A.

#### Eyetracking setup

Six 2.5-inch AprilTags were pasted on the borders of the screen to define planar surfaces and track areas of interest (AOI). Calibration was done, and repeated (if necessary) until data confidence was nearly 100% and accuracy was within 1.5 to 2.5 degrees.

### Measures and procedure

**Fig. 1:**
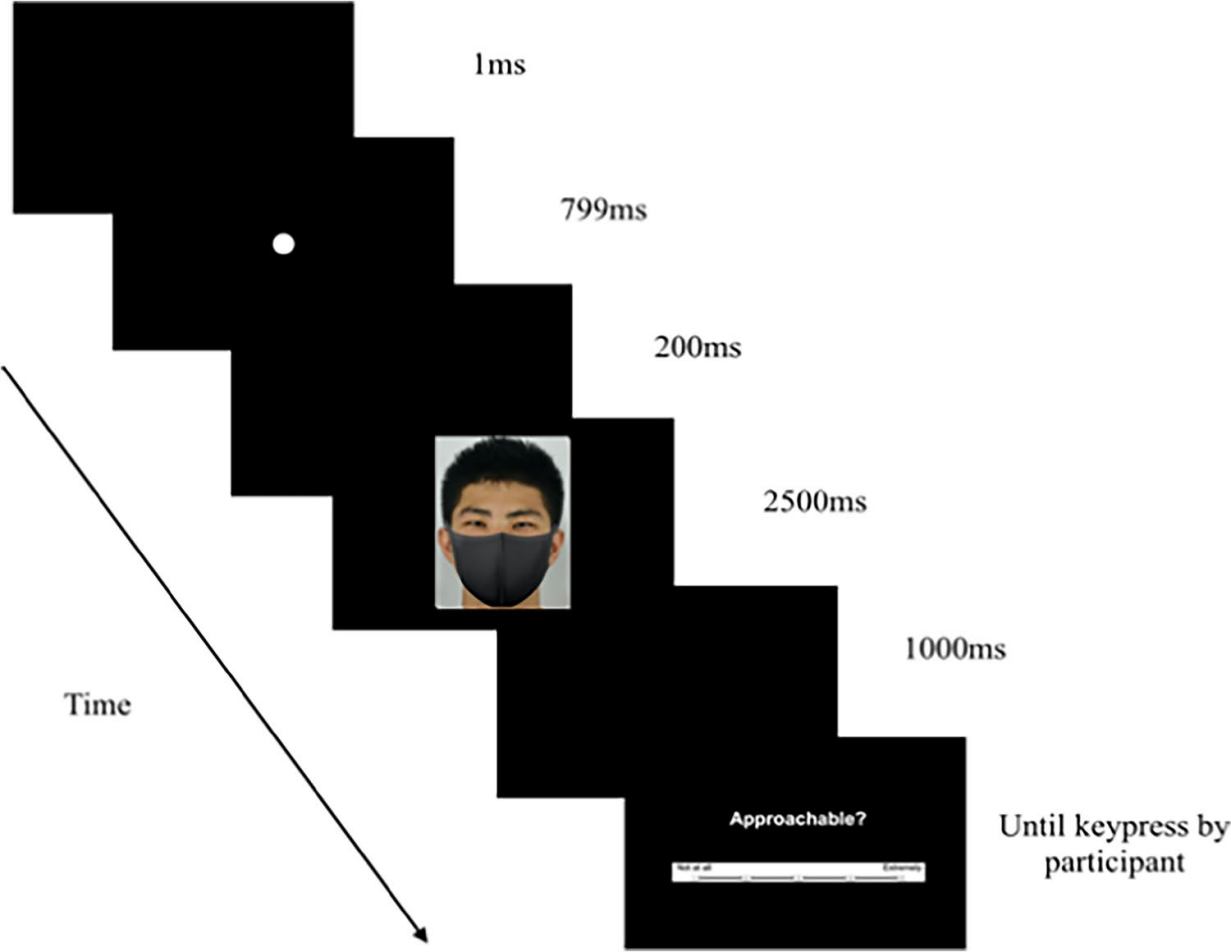
A Sample of trial sequence

After welcoming participants, they were left alone in a cubicle. All instructions were presented on screen. Our procedures were similar to Hermens et al. (2018). There were three blocks of trials, i.e., trustworthiness, approachability, and gaze intensity (Hermens et al., 2018); the block order was randomised. On each trial (see Fig. 1), a white fixation circle (50 by 50 pixels) was presented in the center of the black screen for 800 ms, followed with a blank black screen for 1000 ms. Thereafter, one face randomly chosen from our 60 face stimuli (30 masked and 30 unmasked) was presented for 2,500 ms (see also Eisenbarth & Alpers, 2011) followed by another blank black screen for 1000 ms. Next, a 5-point scale (e.g., “Approachable?”) with anchors 1 (*not at all*) to 5 (*extremely*) was presented. Participants entered a number using their keyboard; no mouse cursor was on screen throughout, thus preventing any visual distractions. At the end of each block, participants took a 30-s break. This was followed by a recalibration of the eye tracker.

 Lastly, participants were thanked and debriefed. Covid-19 safety measures implemented when the experiment was run (March to May 2021), including masking of both participants and the experimenter.

### Post-processing eye-tracking data

Two forms of eye-tracking data were captured. The first form was the video captured from the first-person view, and the second form was the normalized x–y positions^1^ from the eye camera. The integration of these two forms of data was used to derive the eventual time-tagged area-of-interest (AOI) data.

For each trial, two regions of AOIs (see OSM: B) were defined using the Pupil Labs software – the eye region and the masked-up “mouth” region. For each trial, the defined AOI surfaces was then synchronized with the stimuli onset/offset parameters to compute the fixation duration and count.

#### Fixation and duration

The start and end of each stimulus presentation was synchronized with the first-person view captured from the world camera. For any particular face, once the AOI surface definition for that particular face stimulus was applied, the fixation duration and count in respect of the AOI would be computed by the software.

### Inspecting the quality of eye-tracking data

No trials were dropped. All except two participants’ pupil detection confidence, computed by Pupil Lab software, was always close to 100%. That one participant was excluded because 63% of his eye-fixation data was missing, likely due to vision problems although equipment failure could not be ruled out. The other participant was included as there was fixation captured on the face stimuli for all three blocks of face-rating trials, although his/her pupil detection confidence was 90%, much lower than the rest (nearly 100%). In addition to these two participants, we had considered removing the data of two other participants came with glasses^2^ but we eventually retained their data as their eye-tracking data was close to 100% confidence.

## Results

Unlike eyetracking data, the trustworthiness, approachability and gaze intensity were susceptible memory effects. That is, participants could have simply repeated their responses across blocks**,** despite us having randomized the blocks and stimuli within blocks. To rule out this possibility, we computed the correlation and within-participant mean differences for each face across the blocks. For each statistical test, for each mask type, we have 90 p-values (30 faces × 3 comparisons: Block 1 vs. Block 2, Block 2 vs. Block 3, Block 1 vs. Block 3). As shown in Table 1 below, the error rate by chance fell below 5% (the Type I error rate) for most analyses, and was only “marginally” significant at 5.5% for one of the analysis. Together this implies that any memory effects were extremely unlikely.

**Table 1:** Investigating memory effects: Did participants merely repeated their ratings across blocks?

Face Type	Number of significant t-tests	Number of significant correlations
Masked	5/90 (5.5%)	3/90 (3.3%)
Unmasked	1/90 (1.1%)	3/90 (3.3%)

For all subsequent analyses, we adjusted our alpha level to *p* = 0.016 (i.e., 0.05/3) to account for multiple comparisons between approachability, trustworthiness, and gaze intensity conditions.

### Social judgements and gaze cue

**Fig. 2:**
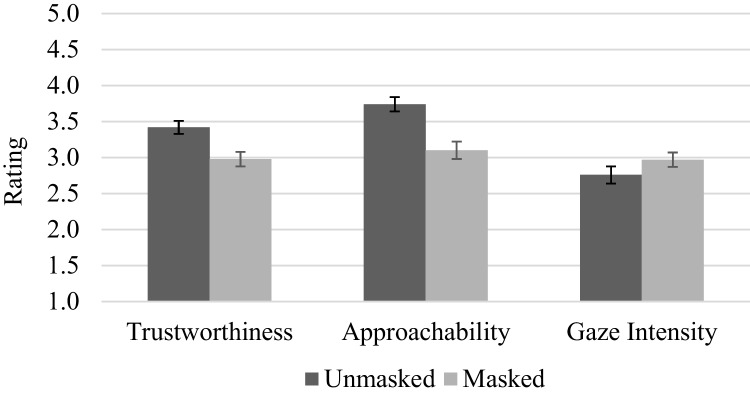
Mean social judgements and perceived gaze intensity ratings. Note. Error bars depict standard error of the mean

Participants rated masked faces as less trustworthy, *t*(36) = -4.21, *p* < 0.001, *d* = -0.69, than unmasked faces. Similarly, participants rated masked faces as less approachable, *t*(36) = -6.65, *p* < 0.001, *d* = -1.09 than unmasked faces. However, the perceived gaze intensity between masked and unmasked faces were similar, *t*(36) = 1.78, *p* = 0.083, *d* = 0.29. See Fig. 2.

Perceived gaze intensity was also not found to be a mediator for the effect of masked condition on both social judgements, because the indirect effects on trustworthiness, *B* = -0.04*,* 95% CI [-0.22, 0.07], and on approachability, *B* = 0.01*,* 95% CI [-0.06, 0.05], were both nonsignificant.

#### Gaze behaviour

##### Eye region AOI

The fixation duration and fixation count across all three blocks were aggregated. Overall, participants looked longer, *t*(36) = 7.69, *p* < 0.001, *d* = 1.26, and looked more frequently, *t*(36) = 7.44, *p* < 0.001, *d* = 1.22, at the eye region of masked faces than unmasked faces.

**Fig. 3:**
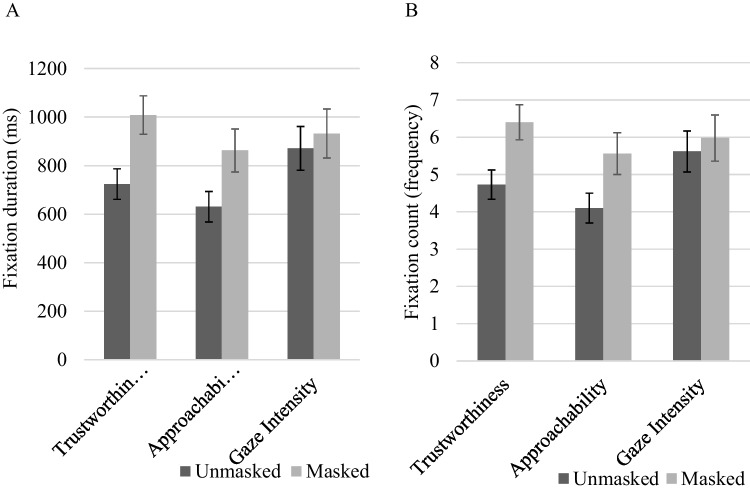
**A** Mean fixation duration (Eye Region AOI). **B** Mean fixation count (Eye Region AOI). Note. Error bars depict standard error of the mean

Subsequently, we split the analysis for each trial type (i.e., trustworthiness, approachability, gaze intensity) and performed pairwise comparisons between masked and unmasked faces. As shown in Fig. 3, when asked to make judgments of trustworthiness and approachability, participants looked longer [trustworthiness: *t*(36) = 7.75, *p* < 0.001, *d* = 1.27; approachability: *t*(36) = 5.13, *p* < 0.001, *d* = 0.84] and more frequently [trustworthiness: *t*(36) = 6.77, *p* < 0.001, *d* = 1.11; approachability: *t*(36) = 4.98, *p* < 0.001, *d* = 0.82] at masked faces as compared to unmasked faces. However, when asked to make judgments of gaze intensity, there was no significant differences in fixation duration, *t*(36) = 1.95, *p* = 0.058, *d* = 0.32, and fixation count, *t*(36) = 1.93, *p* = 0.061, *d* = 0.32, between masked and unmasked faces.

##### Masked region AOI

The masked-up region was important to consider because this region obscured the lips/mouth, an important part of the face during social interaction (Calvo et al., 2018; Hermens et al., 2018; Sutherland et al., 2015; Willis et al., 2011a). Hence, we performed separate pairwise comparisons for masked region AOI, but only for the trustworthiness and approachability block as gaze intensity occurs only when an eye contact was first made to detect another’s gaze (Cavallo et al., 2015; Cui et al., 2019). Note that the masked region of masked and *un*masked faces refer to exactly the same area (see OSM: B).

**Fig. 4:**
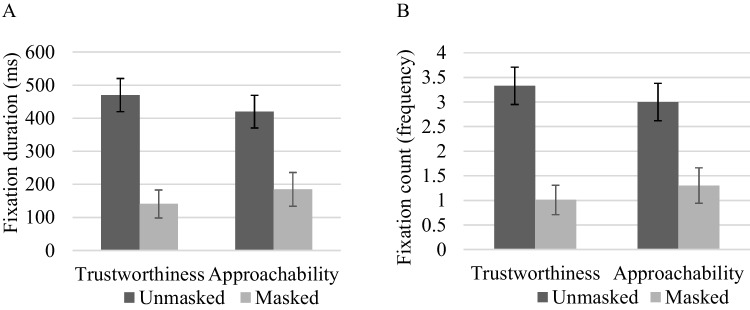
**A** Comparison of mean fixation duration (Masked Region AOI). **B** Comparison of mean fixation count (Masked Region AOI). Note*.* Error bars depict standard error of the mean

**Fig. 5:**
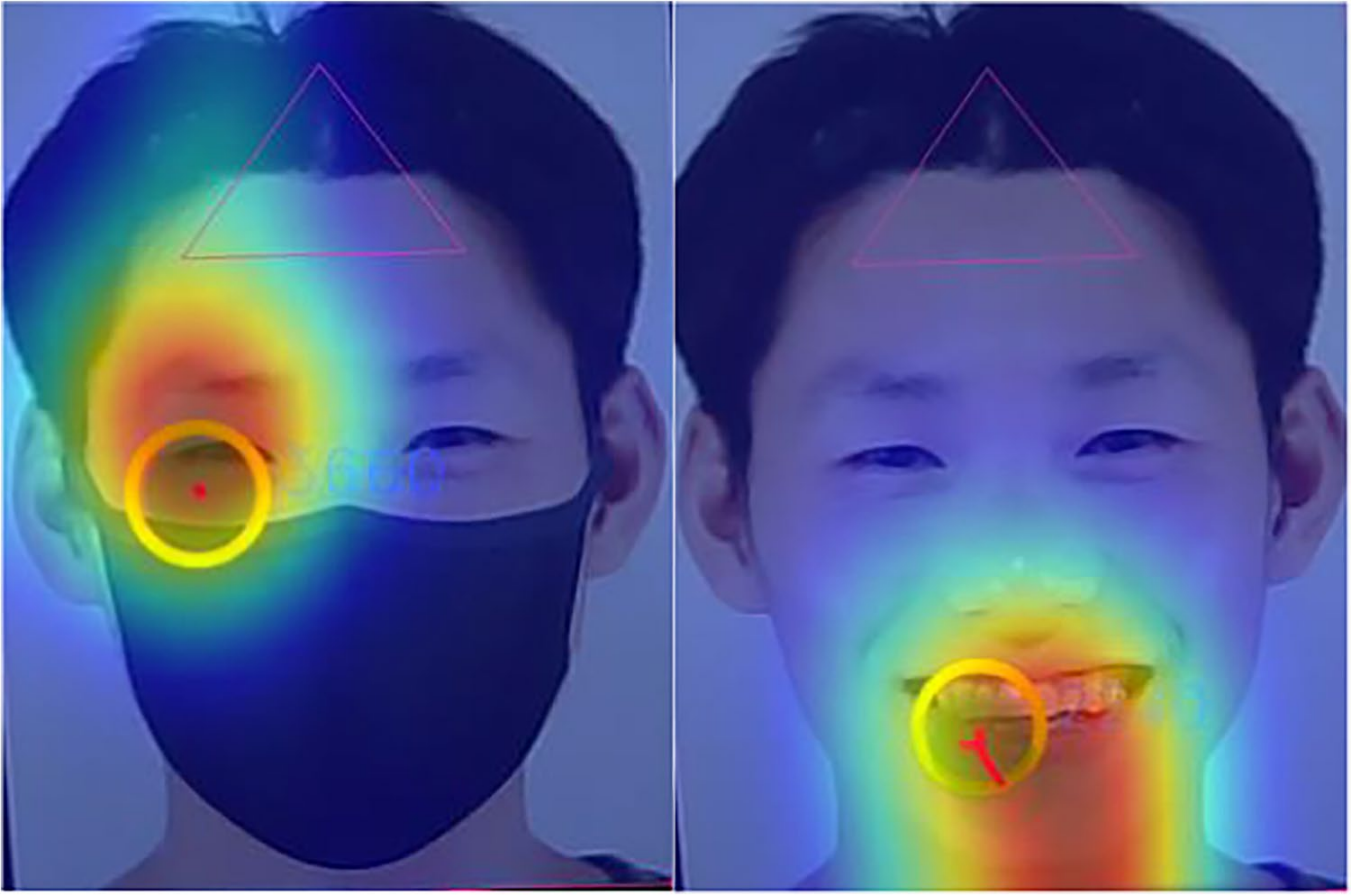
Sample gaze heatmaps. Note*.* Sample heatmaps of fixation duration across masked and unmasked face stimuli with difference in maps showing where more fixations were made between the eye region AOI (for masked face) and the masked region AOI (for unmasked face)

Results showed that in contrast to eye region AOI, when asked to make judgments of trustworthiness and approachability, participants dwelled longer [trustworthiness: *t*(36) = 10.03, *p* < 0.001, *d* = 1.65; approachability: *t*(36) = 4.98, *p* < 0.001, *d* = 0.82] and more frequently [trustworthiness: *t*(36) = 10.18, *p* < 0.001, *d* = 1.67; approachability: *t*(36) = 5.15, *p* < 0.001, *d* = 0.85] on the masked region AOI of *un*masked faces, compared to masked faces, as shown in Fig. 4. Notice that these results were completely the opposite as compared to the eye region AOI analyses (see Fig. 5 for sample gaze heatmaps).

##### Eye region relative to masked region fixation on the same face

**Fig. 6:**
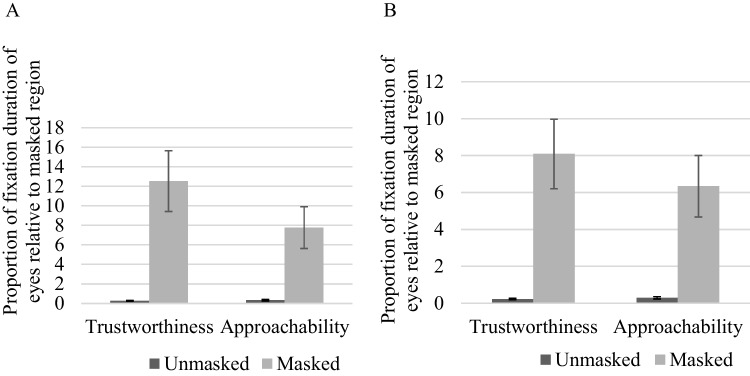
**A** Proportion AOIs fixation duration. **B** Proportion AOIs fixation count. *Note.* Error bars depict standard error of the mean

Given the importance of the proportion of attention (fixation duration and fixation count) allocated to eye versus mouth region (Batty, 2020; Calvo et al., 2018; Eisenbarth & Alpers, 2011; Sutherland et al., 2015; Willis et al., 2011a, b), we performed a pairwise comparisons for the proportion AOIs (eye region relative to masked region) fixation (duration and count) on the same face between unmasked and masked faces. The results showed that masked faces captured significantly more attention on the eye region relative to the masked region, as compared to unmasked faces, when making judgements for both trustworthiness [duration: *t*(36) = 3.96, *p* < 0.001, *d* = 0.65; count: *t*(36) = 4.19, *p* < 0.001, *d* = 0.69] and approachability [duration: *t*(36) = 3.51, *p* = 0.001, *d* = 0.58; count: *t*(36) = 3.64, *p* = 0.001, *d* = 0.60], as shown in Fig. 6.

#### Mediation analyses

The subsequent mediation analysis aimed to explain whether masked faces were judged less trustworthy and less approachable because people automatically paid attention to the nondiagnostic eyes in masked faces. MEMORE within-subjects simple mediation analysis (Montoya, 2018; Montoya & Hayes, 2017) Model 1 was applied, with trustworthiness and approachability ratings as the dependent variables and the proportion of attention (duration and count) spent between the eyes relative to the masked^3^ region (primarily the mouth region) as the potential mediators. Proportion AOIs is a commonly used measure in eye-tracking studies when comparing fixations between AOIs of the same face in determining face evaluation outcomes (Adams & Kleck, 2005; Eisenbarth & Alpers, 2011; Willis et al., 2011b).

**Fig. 7:**
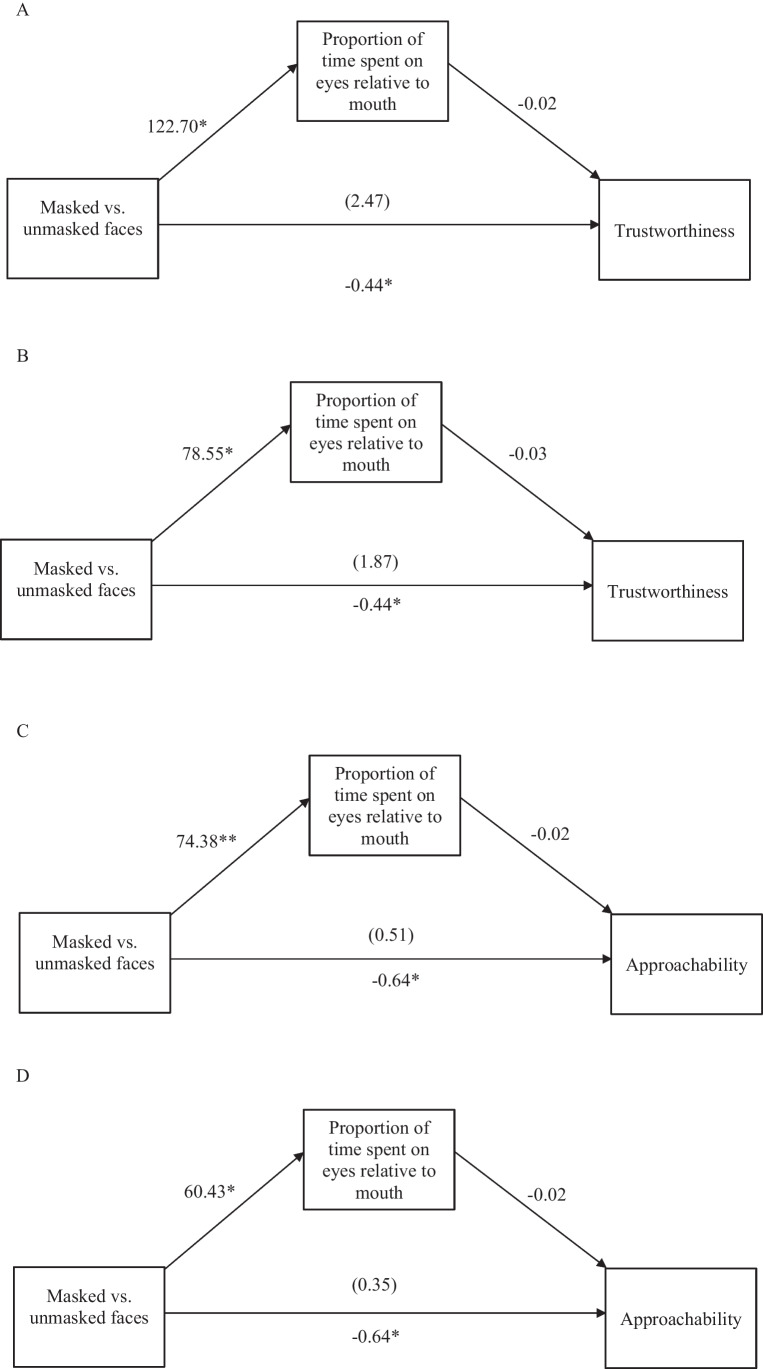
**A** Diagram of the simple mediation model 1 using MEMORE for trustworthiness (Duration)^a^. **B** Diagram of the simple mediation model 1 using MEMORE for trustworthiness (Frequency). **C** Diagram of the simple mediation model 1 using MEMORE for approachability (Duration)^a^ **D** Diagram of the simple mediation model 1 using MEMORE for approachability (Frequency)^a^. *Notes*: ^a^Under MEMORE mediation analysis for repeated measures, the inference for mediation effect is the coefficient of ab (indirect effect), being deemed different from zero, instead of relying on the causal steps that the individual components a and b must be significant before establishing mediation has occurred — this is in recognition that the mediation quantifies the effect of interest rather than a combined hypothesis test of a and b (Montoya & Hayes, 2017). The values are the unstandardised beta coefficients (B) of the various pathways. The direct effect of IV on DV is listed on the top in the parenthesis and the total effect is listed at the bottom. *p < 0.001. **p = 0.001

There were four sets of mediation analyses (social judgment: approachability vs. trustworthiness; mediator: fixation duration vs. fixation count). The number of bootstrap samples was set at 5000. When making trustworthiness judgments (see Fig. 7A and B), while masked faces led people to look longer at the eyes relative to the masked region, *B* = 122.70*, SE* = 30.96*, p* < 0.001, the rating was not significantly different from unmasked faces, with nonsignificant indirect effect, *B* = -2.91*,* 95% CI [-9.91, 1.47]. Similarly, masked faces led people to look more frequently at the eyes relative to the masked region, *B* = 78.55*, SE* = 18.73, *p* < 0.001, but did not in turn lower trustworthy judgments, with a nonsignificant indirect effect, *B* = -2.31*,* 95% CI [-6.80, 0.25].

When judging approachability (see Fig. 7C and D), masked faces led longer fixation at the eyes relative to the masked region, *B* = 74.38*, SE* = 21.20*, p* = 0.001, and this in turn lowered how approachable one judged the target, with a significant indirect effect, *B* = -1.15, 95% CI [-4.48, -0.19]. Similarly, masked faces led people to look more frequently at the eyes relative to the masked region, *B* = 60.43*, SE* = 16.59*, p* < 0.001 and this in turn lowered how approachable one judged the target, with significant indirect effect, *B* = -0.99*,* 95% CI [-3.92, -0.17].

Altogether, the mediation analyses suggests that masked faces may lead people to look at others’ eyes for affiliative, yet nondiagnostic, social cues of approachability (but not trustworthiness) in the eyes.

## Discussion

The compensatory hypothesis was thus not supported; masked happy faces did not have sufficient enhanced (or more intense) gaze cue effect that could make them appear more approachable or trustworthy. Rather, the inhibitory hypothesis was supported; as people focused more on the eye region relative to the mouth region of masked happy faces, the faces were rated lower in approachability, even as trustworthiness judgment was unaffected. More importantly, we found evidence for mediation, which previous similar studies did not (Cartaud et al., 2020; Grundmann et al., 2021; Lau, 2021; Marini et al., 2021). The direction of the mediation analyses of our eyetracking data gave us a unique insight into the phenomenon: When people view others who are masked up, they spontaneously look at others’ eyes but not necessarily for affiliative cues.

### Diagnostic facial cue for approachability and trustworthiness

The fact that masked happy faces with obscured smiles were judged as less approachable and less trustworthy is consistent with prior studies demonstrating the importance of facial expression, i.e., happiness or smiling, as cue signaling approachability and trustworthiness (Calvo et al., 2018; Dzhelyova et al., 2012; Sutherland et al., 2015; Willis et al., 2011a). Unmasked faces with greater fixation around the otherwise masked-up region, i.e., primarily the smiling mouth area, being rated more approachable and trustworthy further suggested that it is the obscured facial expression cue on masked faces, instead of eye gaze, that is likely diagnostic for these two social judgements. While prior studies found the eye region, including the eyebrows, as one of the critical cues for social judgements, particularly trustworthiness (Calvo et al., 2018; Eisenbarth & Alpers, 2011; Hermens et al., 2018; Oosterhof & Todorov, 2008), the current findings did not support the eye region as diagnostic cue for approachability and trustworthiness perhaps due to the lack of enhanced gaze effect coming from static images (Frith, 2009; Hamilton, 2016; Krämer et al., 2014; Vicaria et al., 2015).

When viewing unmasked faces where a smile is clearly visible, people pay more attention to the mouth region compared to the eye region, and this led to an increase in regarding these faces as more approachable. This is in line with prior studies establishing that approachability judgement relies on broad cues such as smile, or facial expression of happiness, as inference of approach or avoidance behavioral intent (Sutherland et al., 2015; Willis et al., 2011a). While gaze signals an intent to communicate (e.g., Adams & Kleck, 2005), it pales in comparison to the smiling mouth region, having greater positive effect on approachability rating as demonstrated in the mediation analysis outcome. Mask-wearing thus inhibits this critical cue for judging approachability.

In contrast, trustworthiness judgements do not rely on a distinct facial region as diagnostic cue, as shown in prior studies (Eisenbarth & Alpers, 2011; Hermens et al., 2018), but relied primarily on the congruency between the various cues such as eye gaze accompanied by inverted-V shaped eyebrows and a smiling mouth (Calvo et al., 2018; Dzhelyova et al., 2012; Kaisler & Leder, 2017). Past eye-tracking studies showing saccadic eye movements across the facial regions when judging trustworthiness (Peterson & Eckstein, 2012) also indicated that people must be integrating information from various facial cues, which possibly explains the lack of mediation effect of higher masked region fixation (relative to eye region). Nevertheless, the findings suggested that the masked-up region remains a critical missing cue for trustworthiness judgement given the higher trustworthiness rating on unmasked faces with more dwell time and frequency on that region albeit masked faces had a higher eye region fixation.

Together, the findings from the present study demonstrated that the masked-up region, i.e., mainly the mouth region, is a more diagnostic cue for approachability and trustworthiness judgements than the exposed eye region when judging happy faces.

### Limitations and implications for future research

One limitation of our study is the use of only happy faces to test the inhibition hypothesis. It remains an open question whether our results would generalize if other facial expressions were used. A particular case could be argued for anger, where the eye region (eyebrows pulled down and together, eyes opened wide; Sayette et al., 2001) is a diagnostic feature that is unobscured – and possibly unaffected – by face masks. In other words, one can tell if another person is displaying anger or not irrespective of whether that person is wearing face masks. Thus, it might be that when asked to make trustworthiness and approachability judgments of angry faces, there may be no difference between masked versus unmasked faces, unlike what is found here when happy faces were used. A recent study (Twele et al., 2022) had explored this to a certain extent with a focus on trustworthiness; further work is still needed particularly on approachability.

Another limitation is the use of static facial stimuli, which does not dissimilar to real-life social interaction. Future study could explore using dynamic faces (e.g., Jack et al., 2014) to improve external validity.

Future studies could also investigate the effect of time and culture on judging masked faces. With mask-wearing policy being implemented for some time in many countries, seeing masked faces has gradually become a social norm which could enhance acceptance (Carbon, 2021), albeit resistance could remain (Lang et al., 2021). Hence there is a possibility that social judgements of masked faces could change (or have changed) over time as well. Longitudinal studies comparing the social judgements ratings of masked vs. unmasked faces across countries or cultures with varying mask-wearing acceptance/resistance levels could shed further light to what had been established in the current study. Considering the importance of the eye region when forming social judgements, particularly trustworthiness, across different cultural groups (Mo et al., 2022), the effect of possible cultural differences in the perception of eye contact (Akechi et al., 2013) when judging masked faces could also be explored.

### Conclusions and reflections

The ability to make accurate inferences from cues of others’ behavioral intent is a key element of social interaction in order to prepare an appropriate behavioral response. Mask-wearing inhibits the critical facial cues needed for social interactions and has the potential to discount how approachable or trustworthy one truly is and could trigger undesired behavioral response such as avoidance and mistrust which result in hefty interpersonal costs. Although wearing face masks during the Covid-19 pandemic benefits public health, the impact on social interactions may have been underappreciated.

## Supplementary Information

Below is the link to the electronic supplementary material.

Supplementary file1 (DOCX 746 KB)

## Data Availability

Data is available at https://osf.io/zekw2/.

## Footnotes

1 The reason why x–y is normalized is because everyone’s screen sizes are different, so normalization is a way of making sure the eye-tracking data is accurately tied to our screen resolution.

2 Technically, the eye-tracker could be worn over participants’ glasses. However, the ultraviolet coating of their glasses may interfere with the infrared light emitted from the eye camera. That is why, during recruitment, bespectacled participants were told not to come with glasses but with contact lens instead.

3 “Masked” region to be treated as synonymous with the “mouth” region in Fig. 7A to D for ease of reference.
