# Supplementary material for: Face masks inhibit facial cues for approachability and trustworthiness: an eyetracking study
Source: Curr Psychol. 2022 Oct 6:1–12. Online ahead of print. doi: 10.1007/s12144-022-03705-8 (PMC9535231; doi:10.1007/s12144-022-03705-8)
Supplement: Supplementary file 1 — Supplementary file1 (DOCX 746 KB) [file 12144_2022_3705_MOESM1_ESM.docx]

**Online Supplementary Materials (OSM): A**

Below were the face stimuli that were used in our experiment. The faces contained no accessories or colored makeup that could potentially bias the perception of their age, expression or related skin texture. The references (e.g., “Y14F-21_happy”) are original filenames used in Tsinghua-FED (Tsinghua University, 2020). In this example, Y14F-21_happy means young female (photo #14) who is 21-years-old and has a happy expression. A face mask was then superimposed onto the original face.

*
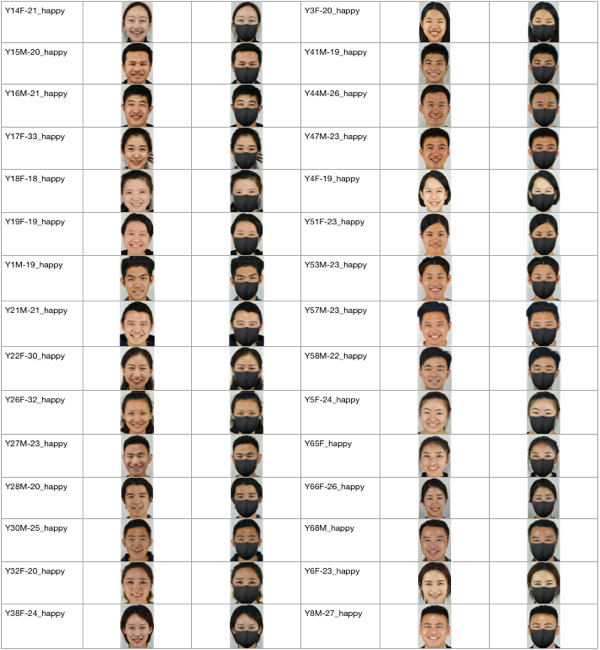
*

**Online Supplementary Materials (OSM): B**

Sample AOIs are displayed below. AOIs appear as pink boundary shapes. Left column shows the sample eye region AOI for unmasked (top) and masked (bottom) face stimuli. Right column shows the sample masked region AOI for masked (top) and unmasked (bottom) face stimuli.

| 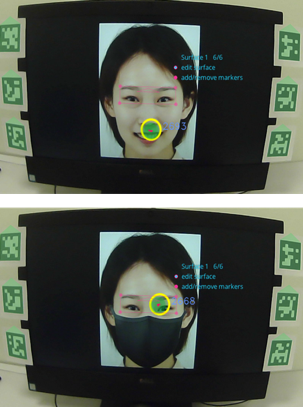 | 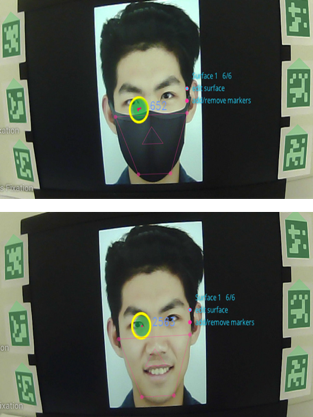 |
| --- | --- |
